# Virulence reduction in bacteriophage resistant bacteria

**DOI:** 10.3389/fmicb.2015.00343

**Published:** 2015-04-23

**Authors:** Marcela León, Roberto Bastías

**Affiliations:** Laboratory of Microbiology, Institute of Biology, Pontificia Universidad Católica de ValparaísoValparaíso, Chile

**Keywords:** bacteriophage, bacteriophage resistance, virulence change, bacteriophage receptor

## Abstract

Bacteriophages can influence the abundance, diversity, and evolution of bacterial communities. Several bacteriophages have been reported to add virulence factors to their host and to increase bacterial virulence. However, lytic bacteriophages can also exert a selective pressure allowing the proliferation of strains with reduced virulence. This reduction can be explained because bacteriophages use structures present on the bacterial surface as receptors, which can be virulence factors in different bacterial species. Therefore, strains with modifications in these receptors will be resistant to bacteriophage infection and may also exhibit reduced virulence. This mini-review summarizes the reports on bacteriophage-resistant strains with reductions in virulence, and it discusses the potential consequences in phage therapy and in the use of bacteriophages to select attenuated strains for vaccines.

## Introduction

Bacteriophages are likely the most abundant microorganisms on earth (Fuhrman, 1999). They can affect the evolution of bacteria through lysogenic conversion and transduction (Canchaya et al., 2003), and they can exert selective pressure due to their lytic effects (Middelboe et al., 2001; Shapiro et al., 2010). The role of bacteriophages in increasing bacterial virulence has been widely studied (Wagner and Waldor, 2002; Brussow et al., 2004). However, the effect of bacteriophages on the reduction of bacterial virulence is still poorly understood and has not been reviewed.

Although bacteria can have mechanisms to resist phage infection, such as the Restriction Modification System (Tock and Dryden, 2005) and the CRISPR-Cas system (van der Oost et al., 2014), the proliferation of bacteriophage resistant (BR thereafter) strains is a natural process associated with spontaneous mutations and adaptation, frequently related to modifications in bacteriophage receptors (Labrie et al., 2010). Virtually any structure on the cell surface can be a receptor, and its recognition by bacteriophage proteins determines bacteriophage specificity and host range (Hyman and Abedon, 2010; Rakhuba et al., 2010). These structures can be classified according to their structural characteristics, and they have several roles, including acting as virulence factors (Koebnik et al., 2000; Raetz and Whitfield, 2002; Boneca, 2005; Proft and Baker, 2009; Brown et al., 2013). Modifications in bacteriophage receptors will confer resistance to bacteria; however, this resistance could have a secondary cost for the bacteria. The modifications may result in lower fitness compared to non-resistant strains. According to several reports (**Table 1**), bacteriophage resistance can also be related to lower virulence, making the resistant bacteria less virulent than the non-resistant ones (Heierson et al., 1986; Park et al., 2000; Santander and Robeson, 2007; Capparelli et al., 2010b; Friman et al., 2011; Laanto et al., 2012). This review discusses the association between bacteriophage resistance and virulence reduction in bacteria due to modifications in bacteriophage receptors, and it discusses the potential implications of this phenomenon.

**Table 1:** Published results relating bacteriophage resistance to bacterial virulence reduction.

Bacterium	Phage	Primary results	Virulence factor affected	Reference
*Pseudomonas plecoglossicida*	PPpW-3, PPpW-4	The bacteriophage resistant (BR) strains present lower virulence (LD_50_ > 10^4^ CFU fish ^-1^) than the parental strains (LD_50_ 10^1.2^ CFU fish^-1^) in ayu.	ND	Park et al. (2000)
*Salmonella enterica* serovar Enteritidis	*f*2αSE, *f*3αSE, *f*18αSE	The BR strains lack the *O*-polysaccharide and are avirulent against *Caenorhabditis elegans*.	Polysaccharide *O* (O-PS)	Santander and Robeson (2007)
*Salmonella enterica* serovar Parathyphi B	Φ1	The BR strains are avirulent and protect mice against lethal doses of *Salmonella enterica* serovar Paratyphi B and other serovars of *Salmonella enterica.*	O-Antigen	Capparelli et al. (2010a)
*Serratia marcescens*	PPV	In the absence of phages, high temperature increases the motility and virulence of *S. marcescens*. In the presence of phages, the resistant bacteria have reduced virulence.	ND	Friman et al. (2011)
*Flavobacterium columnare*	FCL-1, FCV-1, FCL-2	The BR strains have reduced virulence in zebrafish, are non-motile and form colonies with abnormal morphology.	ND	Laanto et al. (2012)
*Bacillus thuringiensis* subspecies gelechiae	Φ31, Φ41 Φ42, Φ43 Φ51, Φ52 Φ63	The BR strains have reduced virulence in *Hyalophora cecropia* and are sensitive to methicillin.	ND	Heierson et al. (1986)
*Staphylococcus aureus*	M^Sa^	The BR strain has a reduced growth rate, under-expression of several virulence factors, produces a capsular polysaccharide, and can protect mice against infection by methicillin resistant *Staphylococcus aureus* (MRSA) and vancomycin-intermediate (VISA) strains of *S. aureus*.	Terminal *N*-acetylglucosamine from wall teichoic acids	Capparelli et al. (2010b)

## Bacteriophage Receptors in Gram-Negative Bacteria

The Gram-negative cell wall comprises several layers, with an outer membrane that differs from the membranes present in Gram-positive bacteria. The external layer of the outer membrane is composed of lipopolysaccharide (LPS), which is present only in Gram-negative bacteria and is associated with endotoxicity. There are several proteins, called outer membrane proteins (OMPs), that are embedded in the membrane and participate primarily in transport and diffusion mechanisms (Koebnik et al., 2000). Both LPS and OMPs have been related to virulence in various Gram-negative bacteria (Raetz and Whitfield, 2002; Bugla-Ploskonska et al., 2007), and they are also bacteriophage receptors, which in some cases are necessary simultaneously for adsorption (Rakhuba et al., 2010).

### Lipopolysaccharide

Lipopolysaccharide is composed of lipid A and polysaccharides. This molecule is a potent endotoxin, and its role in virulence has been widely discussed (Raetz and Whitfield, 2002; Trent et al., 2006). Variations in the lipid A of three pathogenic bacteria from the genus *Yersinia* produce alterations in virulence (Rebeil et al., 2004); Likewise, the genes *wbaP*, *wzy*, and *wzz* participate in *O*-antigen synthesis and are necessary for human serum resistance to *Salmonella enterica* serovar Typhi (Hoare et al., 2006). LPS is also a receptor for numerous bacteriophages (Ishiguro et al., 1983; Michel et al., 2010; Kiljunen et al., 2011). Direct interaction between phage proteins and LPS has been demonstrated, including by X-ray crystallography evidence (Steinbacher et al., 1997; Inagaki et al., 2000).

Chart et al. (1989) showed that the loss of LPS in *Salmonella* produces changes in bacteriophage susceptibility and the loss of virulence in mice. More recent work has shown that strains of *Salmonella enterica* serovar Enteritidis resistant to the lytic phages *f2α*SE, *f3α*SE, and *f18α*SE are avirulent in the nematode *Caenorhabditis elegans*. The BR strains show abnormal colony morphology and lack the *O*-polysaccharide from LPS, suggesting that modifications in this structure may be involved in resistance and the loss of virulence (Santander and Robeson, 2007). A similar situation was observed with the strain Salp572^ϕ1S^ of *Salmonella enterica* serovar Paratyphi B and bacteriophage ϕ1 (Capparelli et al., 2010a). A BR strain called Salp572^ϕ1R^ lacks the *O*-polysaccharide from LPS, and gene expression analysis has revealed that Salp572^ϕ1R^ under-expresses several genes related to virulence, such as *cmE, sthE,* and *cheY* (periplasmic heme-dependent peroxidase, a putative major fimbrial subunit, and a chemotaxis regulator, respectively). Moreover, Salp572^ϕ1R^ was completely avirulent in mice; whereas the parental sensitive strain Salp572^ϕ1S^ killed the mice after 48 h of infection. These examples strongly suggest that modifications in LPS can produce bacteriophage resistance and decrease virulence simultaneously.

### Outer Membrane Proteins

Outer membrane proteins have several functions in the cell (Koebnik et al., 2000; Bugla-Ploskonska et al., 2007). These proteins have also been related to virulence in *Listonella anguillarum*, *Helicobacter pylori*, *Escherichia coli*, *Neisseria meningitidis*, *Salmonella Typhimurium,* and several *Vibrio* species (Yamaoka et al., 2002; Bugla-Ploskonska et al., 2007; Naka and Crosa, 2012). In *L. anguillarum,* five OMPs are related to osmolarity regulation, which occurs when the bacteria move from seawater to lower salinity environments in the host (Kao et al., 2009). In *E. coli,* the proteins OmpW and OmpA participate in immune system evasion (Smith et al., 2007; Wu et al., 2013).

Outer membrane proteins are recognized receptors for numerous bacteriophages, including the bacteriophage Lambda, whose receptor is the protein LamB from *E. coli* (Chatterjee and Rothenberg, 2012). BR strains with mutations in OMPs have repeatedly been found (Skurray et al., 1974; Inoue et al., 1995). For example, BR strains from *E. coli* K-1 present alterations in the protein sequence of OmpA (Morona et al., 1984), which participates in conjugation, adhesion, and immune system evasion, and is a receptor for several T-even-like bacteriophages (Smith et al., 2007). Although there are no studies on virulence reduction in these strains, it is possible that mutations in OmpA also alter bacterial adhesion or immune system evasion.

## Bacteriophage Receptors in Gram-Positive Bacteria

The Gram-positive cell wall lacks the LPS present in Gram-negative membranes, but it has a thick layer of peptidoglycan interspersed with acid polysaccharides called teichoic acids. In addition to their structural function, these molecules can contribute to virulence by participating in adhesion and toxicity (Boneca, 2005; Brown et al., 2013). These structures are also receptors for several bacteriophages. For example, the infection of *Listeria monocytogenes* by bacteriophages A118 and A500 depends on cell wall teichoic acids, and the broad host range of *Listeria* bacteriophage A511 recognizes only the peptidoglycan (Wendlinger et al., 1996). Similarly, the bacteriophage SPP1 from *Bacillus subtilis* binds to teichoic acids in a reversible manner, and the presence of these polysaccharides accelerates adsorption (Baptista et al., 2008).

Teichoic acids are important for the virulence of *Staphylococcus aureus* because strains lacking this polysaccharide exhibit a reduction in the adherence to human endothelial cells and attenuated virulence in rabbits (Weidenmaier et al., 2005). Likewise, the lack of D-alanine in teichoic acids increases susceptibility to human neutrophils and decreases virulence against mice (Collins et al., 2002). Recent studies performed with the bacteriophage M^sa^ of *S. aureus* showed that a resistant strain called 172 under-expresses several genes related to virulence, such as *sbi* (IgG-binding protein), *ica* (*N*-glycosyltransferase), and a gene for an α-hemolysin. This strain also has a reduced growth rate, and unlike the parental strain, it produces a capsular polysaccharide. The *N*-acetylglucosamine from teichoic acids was identified as a bacteriophage receptor because phage infection was inhibited in the presence of these polysaccharides and because strain 172 lacks *N-*acetylglucosamine. Intramuscular injection with 172 was not lethal, and it was an effective vaccine against infection with other *S. aureus* strains (Capparelli et al., 2010b). These findings suggest that the missing *N*-acetylglucosamine in 172, in addition to its role as a bacteriophage receptor, may also be involved in virulence. However, it is also possible that capsular polysaccharide production confers resistance to bacteriophage infection, as occurs in other bacterial species (Ohshima et al., 1988; Scholl et al., 2005).

## Capsule

The capsule (*K* antigen) is an external structure composed of polysaccharides found surrounding the cells of several bacterial species, and it has been related to adhesion and evasion of the immune system (O’Riordan and Lee, 2004). Bacteria with a capsule, such as *Streptococcus pneumonia,* are more resistant to phagocytosis, and as a consequence, they are more virulent. Mutants deficient in this structure have a significant reduction in their virulence compared to wild type strains (Ricci et al., 2013).

Although the capsule is commonly associated with bacteriophage resistance (Ohshima et al., 1988; Scholl et al., 2005), some bacteriophages can recognize this structure as a receptor, and they have evolved along with the bacteria to recognize different types of *K* antigen. The podophage K1-5 of *E. coli* has two types of tail fiber proteins, which allow it to recognize bacteria with K1 and K5 antigens, and it has two specific glycosidases that enable it to penetrate both types of capsules (Scholl et al., 2001; Leiman et al., 2007). It has been reported that *E. coli* strains lacking the K antigen are insensitive to phage infection (Stirm, 1968). Therefore, if the capsule is recognized as a receptor, the proliferation of BR strains can be associated with virulence reduction due to the participation of this structure in the evasion of the immune system. In this case, the potential virulence reduction in BR strains will depend on the role of the capsule in the specific bacterial species because there are examples of BR strains with alterations in capsule receptors but unaltered virulence (Attridge et al., 2001).

## Bacterial Appendices and Other Factors Associated with Motility

The bacterial flagellum allows bacteria to move into favorable environments and escape from unfavorable environments. In the early stages of infection, the flagellum allows a pathogen to reach the host, and participates in the adhesion process (Josenhans and Suerbaum, 2002). The flagellum has been reported to be an important virulence factor in *Pseudomonas aeruginosa, Vibrio cholerae, Salmonella enterica* serovar typhimurium, *H. pylori* and many species (Eaton et al., 1992; Feldman et al., 1998; Watnick et al., 2001; Josenhans and Suerbaum, 2002; Stecher et al., 2004). Strains of *V. anguillarum* with deletions in the N-terminal region of flagellin A, a major component of the flagellum filament, have reduced motility, and they show reduced virulence in rainbow trout (*Oncorhynchus mykiss*) when the fish is infected through bath treatment, demonstrating the importance of flagella during the first steps of infection (Milton et al., 1996). The flagellum was one of the first bacteriophage receptors identified (Meynell, 1961), and it is now a recognized receptor for numerous bacteriophages (Rakhuba et al., 2010). According to several reports, the flagellum is used as a primary receptor to increase the local concentration of bacteriophages, and it facilitates interaction with a secondary receptor located on the bacterial surface (Schade et al., 1967). However, there are also phages that inject their DNA directly into the flagellum filament (Choi et al., 2013). Thus, it is probable that bacteria with mutations in their flagella-encoding genes will have impaired motility and will be more resistant to bacteriophages that use this structure as a receptor.

Indirect associations between motility, virulence, and bacteriophage resistance were observed by Friman et al. (2011) when they used temperature and bacteriophages as selective pressures to isolate BR mutant strains from *Serratia marcescens* with changes in virulence traits. The isolated mutants had increased motility and virulence against the insect *Parasemia plantaginis*; however, these mutant strains were detected only when the bacteria were grown in the absence of bacteriophages, suggesting an inverse correlation between the presence of bacteriophages and virulence. A clearer link was observed with bacteria from the genus *Flavobacterium,* which exhibit gliding motility. This mechanism of motility involves proteins from the cell surface and does not involve pili or flagella (McBride, 2004). Laanto et al. (2012) showed that BR strains from the fish pathogen *Flavobacterium columnare* present abnormal rough colony morphology and have reduced motility. Moreover, the resistant strains were totally avirulent in zebrafish (*Danio rerio*), while the ancestral strains produced a 25–100% mortality rate. As occurs with the flagella, proteins related to gliding motility are important for virulence (Sato et al., 2010); therefore, it is likely that mutations conferring resistance to bacteriophages in these strains also impair bacterial motility and reduce virulence. A similar situation was observed in BR strains of *B. thuringiensis*: a subset of the resistant strains lack flagella and have reduced virulence in the pupae of the cecropia moth (*Hyalophora cecropia*); however, no direct relationship between bacteriophage resistance and virulence was observed because fully virulent BR strains were also isolated (Heierson et al., 1986).

The pilus is another filamentous appendage that is present in some bacteria, but it is smaller than the flagellum (Proft and Baker, 2009; Melville and Craig, 2013). This structure has been related to virulence and many other functions in bacteria (Silverman, 1997; Mattick, 2002; Proft and Baker, 2009), and it acts as a receptor for many bacteriophages. The most well described case is the bacteriophage ΦCTX of *V. cholearae* (Waldor and Mekalanos, 1996), which carries a gene coding for the cholera toxin (CT) and infects bacteria using a toxin co-regulated pilus (TCP). This pilus is also considered a virulence factor in the bacterium, and both the CT and the TCP are necessary for a fully virulent bacterium. Therefore, in *V. cholerae* strains lacking the TCP receptor or with modifications, the receptor cannot be infected by the ΦCTX phage, and these strains are less likely to increase their virulence.

## Discussion

The main mechanism driving bacteria and bacteriophage co-evolution is spontaneous mutation (Pal et al., 2007; Koskella and Brockhurst, 2014). The random nature of mutations can lead to different phenotypes, including reduced virulence in BR strains. Therefore, the mutation will determine the effect on the bacterium, and it will likely determine the level of virulence reduction. Mutations in regulators of gene expression are generally more likely to result in resistance and virulence reduction because more genes are affected by them. This mechanism may have occurred for strain Salp572^ϕ1R^ of *Salmonella enterica* and strain 172 of *S. aureus* because several genes were found to be under-expressed in both cases (Capparelli et al., 2010a,b). Whether the BR strain with reduced virulence will persist will depend on several factors, such as the presence of bacteriophage, the availability of nutrients and the environmental conditions (Koskella and Brockhurst, 2014). Without the selective pressure of bacteriophages, the BR strains can revert to the parental phenotype, as was observed by Heierson et al. (1986) with *B. thuringiensis,* or the BR stains may be displaced by non-resistant strains with better fitness. Capparelli et al. (2010a) observed that strain Salp572^ϕ1R^ can persist for only a short period of time *in vivo*, likely due to the impairment of virulence factors. However, the same bacterium would have better chances of persisting outside of the host in an environment with predators. Interestingly, a BR strain can become resistant to several bacteriophages simultaneously, as was observed by Santander and Robeson (2007) and others (Betts et al., 2014). This multi-resistance likely occurs because the bacteriophages tested use the same receptor. However, in other cases, the acquisition of resistance against particular bacteriophages can increase susceptibility to other bacteriophages, demonstrating the complexity of this phenomenon in the environment (Marston et al., 2012).

Some of the articles mentioned here describe the use of bacteriophages as antimicrobial agents (Park et al., 2000; Capparelli et al., 2010a), for which the proliferation of resistant bacteria is a major drawback. The results showed that despite BR strain proliferation, the bacteriophage treatments were successful in protecting against bacterial infection; this result was likely due to the virulence reduction in BR strains. Moreover, there are several alternatives to overcome this problem, such as the use of phage cocktails (Loc-Carrillo and Abedon, 2011; Gu et al., 2012). These examples support the use of phages to control pathogenic bacteria; however, this is not a general situation for all phage-bacteria systems because BR strains with unaltered virulence have been isolated previously. Additionally, BR strains of *P. aeruginosa* PAO1 were found to produce higher levels of secreted virulence factors than the parental strain (Hosseinidoust et al., 2013), and bacteriophages can induce a mucoid phenotype in *P. fluorescens,* which is normally associated with virulence in other *Pseudomonas* sp. (Scanlan and Buckling, 2012). Hence, potential alterations in the virulence of BR strains should be determined for every phage-bacteria system, especially if bacteriophages are to be considered for an antimicrobial treatment.

Capparelli et al. (2010b) suggested that bacteriophages could be useful for developing new vaccines due to their ability to select attenuated resistant strains. They showed that BR strain 172 of *S. aureus* was an effective vaccine in mice against lethal doses of different *S. aureus* strains, including methicillin-resistant *S. aureus* (MRSA). The vaccine was effective even when the bacteria were heat-killed, whereas the heat-killed parental strain 170 was not effective. The resistant strain presents a lower growth rate, exhibits under-expression of several virulence genes, and produces extracellular polysaccharide, which, although it is missing in *wt* strains, also has a protective effect in a vaccine. Moreover, this strain modulates the transcription of pro-inflammatory genes (TNF-α, IFN-γ, and IL-1β), and it induces the expression of anti-inflammatory genes (Il-4 and Il-6) in mice. The authors suggest that the use of bacteriophages can facilitate vaccine preparation, allowing the selection of attenuated BR strains. The same authors performed similar work with *Salmonella enterica* serovar Paratyphi B (Capparelli et al., 2010a). In that case, the BR strains were avirulent and proved to be excellent vaccines in mice, supporting the potential use of phages in vaccine development. However, their use should be evaluated carefully in each case by considering the potential of a temperate bacteriophage to increase bacterial virulence (Brussow et al., 2004).

## Conclusion

Bacteriophages can influence the proliferation of new virulent strains. However, although there have been a few reports, the role of bacteriophages in selecting less virulent strains has not been studied in detail. The information presented here suggests that potential changes in the virulence of BR strains depend on the specific bacteriophage-host system. Therefore, this phenomenon must be explored comprehensively, especially if bacteriophages are to be used in phage therapy or in vaccine development (Skurnik et al., 2007; Loc-Carrillo and Abedon, 2011). The cases presented here summarize the information regarding this phenomenon and should be thought of as an incentive to analyze the potential changes in virulence of resistant bacteria every time a phage-bacterial system is investigated.
